# An optotracer-based antibiotic susceptibility test specifically targeting the biofilm lifestyle of *Salmonella*

**DOI:** 10.1016/j.bioflm.2022.100083

**Published:** 2022-09-08

**Authors:** Johannes A. Eckert, Ming Rosenberg, Mikael Rhen, Ferdinand X. Choong, Agneta Richter-Dahlfors

**Affiliations:** a AIMES - Center for the Advancement of Integrated Medical and Engineering Sciences, Karolinska Institutet and KTH Royal Institute of Technology, Stockholm, Sweden; b Department of Neuroscience, Karolinska Institutet, Solnavägen 9, SE-171 77, Stockholm, Sweden; c Department of Microbiology, Tumor and Cell Biology, Karolinska Institutet, Solnavägen 9, SE-171 77, Stockholm, Sweden

**Keywords:** *Salmonella* biofilm, ECM, Curli, Optotracing, EbbaBiolight 680, Real-time, AST

## Abstract

Antimicrobial resistance is a medical threat of global dimensions. Proper antimicrobial susceptibility testing (AST) for drug development, patient diagnosis and treatment is crucial to counteract ineffective drug use and resistance development. Despite the important role of bacterial biofilms in chronic and device-associated infections, the efficacy of antibiotics is determined using planktonic cultures. To address the need for antibiotics targeting bacteria in the biofilm lifestyle, we here present an optotracing-based biofilm-AST using *Salmonella* as model. Our non-disruptive method enables real-time recording of the extracellular matrix (ECM) components, providing specific detection of the biofilm lifestyle. Biofilm formation prior to antibiotic challenge can thus be confirmed and pre-treatment data collected. By introducing Kirby-Bauer discs, we performed a broad screen of the effects of antibiotics representing multiple classes, and identified compounds with ECM inhibitory as well as promoting effects. These compounds were further tested in agar-based dose-response biofilm-AST assays. By quantifying the ECM based on the amount of curli, and by visualizing the biofilm size and morphology, we achieved new information directly reflecting the treated biofilm. This verified the efficacy of several antibiotics that were effective in eradicating pre-formed biofilms, and it uncovered intriguing possible resistance mechanisms initiated in response to treatments. By providing deeper insights into the resistances and susceptibilities of microbes, expanded use of the biofilm-AST will contribute to more effective treatments of infections and reduced resistance development.

## 1 Introduction

Biofilm infections represent a major problem in medicine, primarily as a cause of chronic and device-associated infections [1]. Bacteria adopt a biofilm-specific physiological state when they form aggregates that associate with or adhere to natural or artificial surfaces in the host. The biofilm is signified by a structured consortium of cells surrounded by a self-produced polymer matrix. Compared to planktonic growth, the biofilm mode makes bacteria more resistant to the host's innate and adaptive defence mechanisms, as well as the effects of antibiotics and disinfectants [[2], [3], [4]]. This makes it challenging to treat biofilm infections. The antibiotics currently at hand were developed based on their action on bacteria undergoing planktonic growth, not the biofilm mode. Similarly, antibiotic susceptibility tests (AST) routinely performed in clinical microbiology laboratories worldwide are conducted on planktonically growing bacteria [[5], [6], [7]]. This implies that the antibiotic dosage and therapy prescribed by current ASTs are sub-optimal against biofilm infections, putting the patient at risk of treatment failure or recurrence of the infection.

While sub-optimal use of antibiotics can have severe consequences for the infected individual, it also has global impact as a driver of antimicrobial resistance (AMR) development [8]. Although a complex set of factors cause the spread of AMR, it is generally attributed to excessive use of antimicrobials in both clinical and agricultural settings [9]. If antibiotics become ineffective, common infectious diseases will likely result in prolonged illness, disability, and death. As current projections suggest that resistance will cause ≈300 million premature deaths by the year 2050 [10], there is an urgent need for novel methods that deepen our knowledge on biofilm physiology, composition, and structure. Such information is urgently needed for the development of biofilm-specific antibiotics and adjuvant antibiotics, as well as for the diagnosis and treatment of biofilm infections.

There is an ongoing discussion in the field whether standard methods for microbial biofilm ASTs should be introduced in clinical practice [11]. Realization of such methods greatly depends on access to suitable bioassays. The plethora of techniques available for biofilm studies have been impressively reviewed by Azeredo and colleagues [12]. Many methods are, however, destructive to the biofilm structure, and/or uses dyes toxic to bacteria. While such methods work well for end stage analysis, they are not capable of detecting biofilm formation in real-time. Answering to this need, we recently developed several non-disruptive methodologies based on optotracers, which are non-toxic, optically active fluorescent tracer molecules which when added to the nutrient broth or agar report bacterially produced polysaccharides and peptides in real-time [[13], [14], [15], [16]]. By allowing real-time visualization and quantification of bacterial growth and biofilm development, including formation of the extracellular matrix (ECM), the optotracing methodologies have generated deeper understandings of the formation and composition of *Salmonella* [13,17], *E. coli* [14,17], and *Burkholderia* [18] biofilms.

Herein, we extend our use of optotracers for biofilm analysis by investigating their potential in establishing an AST specific for the biofilm lifestyle. Specific detection of *Salmonella*'s ECM component curli (curli ECM) in real-time [17] suggests a role for the optotracer EbbaBiolight 680 in analysing anti-biofilm activities based on the ECM rather than cellular viability and reduction of total biomass. Catering to the need to generate data of more relevance than planktonic-based assays, thereby improving the prediction of therapeutic success of antibiotics for biofilm infections, we here describe the development of an optotracer-based biofilm-AST.

## 2 MATERIALS and METHODS

### 2.1 Bacterial strains, media, antibiotics, and optotracer

Bacterial strains used in this study include the ISO strain *E. coli* ATCC™ 8739™ [19], *S.* Enteritidis wild-type (wt) strain 3934 [20] as well as strains 3934-p2777 and the isogenic curli mutant 3934 *ΔcsgA*-p2777 [21], which are transformed with the low-copy number plasmid p2777 to enable stable GFP expression [13,22,23]. Bacteria were routinely cultured on Luria-Bertani (LB) agar or in LB broth at 37 °C. To promote *Salmonella* biofilm formation, a salt free formulation of LB (LB w/o salt) agar was used. The antibiotics ampicillin (AMP), oxacillin (OXA), cefoxitin (CXI), vancomycin (VAN), polymyxin B, rifampicin (RIF), ciprofloxacin (CIP), erythromycin (ERY), tetracycline (TET), and gentamicin (GEN) were purchased (Sigma-Aldrich, Stockholm, Sweden) as powder and stored at 4 °C as recommended. Liquid stocks (12.8 mg/mL) were prepared in solvents recommended by the manufacturer: AMP, OXA, CXI, VAN, polymyxin B, CIP, TET, and GEN were dissolved in sterile MiliQ water, RIF in DMSO, and ERY in EtOH. Liquid stocks were filter sterilized through a MF-Millipore™ Membrane Filter, 0.22 μm pore size (Merck, Stockholm, Sweden), and stored at 4 °C until used. The stocks of all antibiotics were serially diluted in MilliQ water to concentrations indicated in the text. The fluorescent tracer molecule EbbaBiolight 680 (Ebba680, Ebba Biotech, Stockholm, Sweden) was stored at 4 °C until used as supplement to the LB agar using 0.5 μl/mL.

### 2.2 Microbroth dilution assay

Microbroth dilution assays were performed in Mueller Hinton II (M − H) broth (Sigma-Aldrich, Stockholm, Sweden) following the protocol described by EUCAST [24]. In the wells of a 96-well plate, each antibiotic was serial diluted in M − H broth to concentrations varying between 1024 and 0.312 μg/mL. Test inoculum of *E. coli* ATCC™ 8739™ and *S.* Enteritidis strain 3934 was prepared by diluting a fresh culture, grown in LB broth to OD_600_ ≈ 0.45, to 5–10 × 10^5^ CFU/mL in M − H broth, from which 50 μL was added to each well. Plates were incubated in a Tecan Infinite M1000 PRO microplate reader (Tecan, Switzerland) at 37 °C for 18 h when absorbance was recorded at 405 nm.

### 2.3 E-tests

E-Tests were performed in 90 mm Petri-dishes containing 25 mL M − H agar, LB agar, or LB w/o salt agar with 4 mm thickness. Using a sterile cotton swab, fresh cultures (OD_600_ ≈ 0.45) of *S.* Enteritidis strain 3934 in LB broth were extensively streaked onto the agar with 90° rotation. E-Test strips (bioMérieux, Sweden) containing AMP (<256 μg/mL), OXA (<256 μg/mL), CXI (<256 μg/mL), VAN (<256 μg/mL), polymyxin B (<1024 μg/mL), RIF (<32 μg/mL), CIP (<32 μg/mL), ERY (<256 μg/mL), TET (<256 μg/mL) and GEN (<256 μg/mL) were placed equally interspaced on the agar such that each plate held 5 strips. Technical duplicates for each antibiotic treatment were included. Plates were incubated at 37 °C or 28 °C for 20 h, after which zones of inhibition were measured.

### 2.4 Optotracing-based biofilm-AST using Kirby-Bauer discs

The biofilm-AST is based on the recently published semi-high throughput optotracing biofilm assay [17]. In each well of a sterile Costar tissue culture treated 6-well plate (Sigma-Aldrich, Stockholm, Sweden), we added 2 mL LB w/o salt agar supplemented with 0.5 μl/mL of the tracer molecule EbbaBiolight 680 (Ebba680, Ebba Biotech AB, Stockholm, Sweden). Plates were allowed to set at room temperature. Plates were inoculated by placing 10 μL of the GFP-expressing *S*. Enteritidis strain 3934-p2777, collected from an exponential phase culture, 5 mm away from the centre of each well. To allow formation of pre-treatment biofilms, plates were incubated for 24 h at 28 °C. Biofilm formation was confirmed by automated fluorescence microscopy, and the physical dimension of biofilms were measured under phase contrast microscopy.

For antibiotic treatment, we used Kirby-Bauer discs (Thermo Fisher, Stockholm, Sweden) containing AMP (10 μg), CXI (30 μg), polymyxin B (300 units), RIF (30 μg), CIP (5 μg), ERY (15 μg), TET (30 μg) and GEN (10 μg), which were stored at 4 °C until use. Blank discs containing no antibiotics were used as mock treatment. Following the formation of pre-treatment biofilms, one antibiotic-containing Kirby-Bauer disc was placed in each well 10 mm away from the centre of the pre-treatment biofilm, plates were then incubated at 28 °C for 72 h. End stage assessment of the biofilm morphology was performed by live automated fluorescence microscopy. GFP was used to locate bacteria, and the relative fluorescence units (RFU) of Ebba680 for visualization and quantification of curli in the ECM. The physical dimension of the biofilm was measured under phase contrast microscopy.

### 2.5 Antibiotic gradient exposure in the optotracing-based biofilm-AST

When preparing agar-containing 6-well plates, we introduced a cylindrical cavity in the agar, 8.5 mm in diameter, by placing an upturned sterile pipette tip in each well of the 6-well plate while adding 2 mL LB w/o salt agar supplemented with Ebba680 (0.5 μg/mL). Once the agar had solidified, tips were removed and the interface between the cavity wall and the bottom of the well was sealed by 10 μL molten LB w/o salt agar added to the cavity. Pre-treatment biofilm formation was initiated using 10 μL inoculum from planktonic cultures of *S.* Enteritidis 3934-p2777 in exponential phase. The inoculum was placed circa 10 mm from the centre of the cavity, then plates were incubated at 28 °C for 24 h. Pre-treatment biofilm formation was confirmed by automated fluorescence microscopy and pre-treatment data collected as previously described. To initiate antibiotic exposure, 80 μL antibiotic solutions were added to the cavities at the following concentrations: CIP (0.125–32 μg/mL), RIF (2–512 μg/mL), CXI (2–512 μg/mL) and polymyxin B (2–512 μg/mL). Plates were incubated at 28 °C for another 72 h before end stage assessment of the biofilm morphology was performed by live automated fluorescence microscopy, using GFP fluorescence for bacterial identification and Ebba680 fluorescence for visualization and quantification of curli in the ECM. The physical dimension of the biofilm was measured under phase contrast microscopy.

### 2.6 Determination of biofilm minimal dose-response inhibition (MD-RIC) and eradication (MD-REC) concentrations

The minimal dose-response inhibition concentration (MD-RIC) of CIP, RIF, CXI, and polymyxin B was determined by analyzing the quantity of curli ECM, measured as RFU from Ebba680 bound to curli, in biofilms treated with different concentrations of the antibiotics.

To determine the minimal dose-response eradication concentration (MD-REC), cell viability after 72 h antibiotic treatment was assessed by collecting the entire biofilm on the agar surface using a sterile loop and resuspend it in 1 mL M − H broth in 24-well plates. Minor homogenization by means of repeated pipetting was performed to moderately disperse the biofilm. Plates were then incubated at 37 °C for 24 h, after which 100 μL of each bacterial suspension was pipetted in quadruplicates into a 96-well plate and OD_405_ was measured in a Tecan Infinite M1000 PRO microplate reader at 405 nm. Eradication of the culture was determined by visual evaluation of turbidity and analysis of OD values. MD-REC was defined as the concentration at which a significant reduction in OD values was observed compared to the mock-treated biofilm.

### 2.7 Estimating minimum bactericidal concentration (MBC) in planktonic cultures

Test inoculum of *S.* Enteritidis strain 3934 was prepared by growing a fresh culture in LB to OD_600 ≈_ 0.45. Following centrifugation, the bacterial suspension was concentrated 10-fold as the pellet was resuspended in LB w/o salt, generating circa 5–10 × 10^9^ CFU/mL. From this suspension, 50 μL was used to inoculate each well of a 96-well plate, in which CIP, RIF, CXI and polymyxin B had been serially diluted in LB w/o salt to concentrations varying between 1024 and 0.312 μg/mL. Wells containing LB w/o salt with no antibiotic were included as mock-treatment control. Following incubation at 37 °C for 24 h, we determined cell viability of each culture by re-inoculating treated bacteria into antibiotic-free medium. This was achieved by transferring 10 μL of each treated culture into a 96-well plate containing 100 μL M − H broth per well. Plates were incubated at 37 °C for 18 h in a Tecan Infinite M1000 PRO microplate reader (Tecan, Switzerland) with OD recorded at 405 nm. Eradication of the culture was determined by visual evaluation of the turbidity and a significant difference between OD values from the treated and mock-treated cultures.

### 2.8 Estimation of CFU in the pre-treatment biofilm

To determine the CFU in pre-treatment biofilms, formed on the agar surface during 24 h incubation, we collected the entire biofilms using sterile 10 μL loops (VWR, Sweden). Each collected biofilm was resuspended in 1 ml PBS in 1.5 ml Eppendorf tubes (Sarstedt. Sweden). To disperse cells of the biofilms, suspensions were homogenized by passage through a 20–200 μL pipette tip (Corning, Sweden) 20 times to ensure that no aggregates were visible. This was followed by vigorous vortexing in 20 s pulses. To confirm proper disaggregation of bacterial aggregates, samples were analysed by optical microscopy. When only single cells were observed, CFU counts of the bacterial suspensions were determined by spread plating on LB agar.

### 2.9 Automated microscopy

Fluorescence imaging of live biofilms (cells and curli ECM) on the agar of the 6-well plates was achieved in a Lionheart™ FX Automated Microscope (Ramcon, Sweden) using the software Gen5 (Ramcon, Sweden). The microscope was loaded with a 1.25× objective, Plan Apochromat WD 5 NA 0.04. GFP was detected using a 465 nm LED cube/GFP filter cube, Ebba680 fluorescence was detected using a 523 nm LED cube/propidium iodide filter cube. The LED intensity, integration time, and gain were 5; 60; 12 for GFP detection, and 5; 600; 10 for Ebba680 detection. Phase contrast was recorded using software pre-settings. Images were acquired under ‘Tiling’ mode, wherein optical signals from the entire well were collected as a 5-by-4 grid that is subsequently ‘stitched’ together during post-imaging analysis. As reference signal for auto-focusing and ‘stitching’, we used GFP fluorescence from bacterial cells in the biofilm.

### 2.10 Spectrophotometric analysis of biofilms

Fluorescence recording of Ebba680 bound to curli was achieved by spectrophotometry using a Tecan Infinite M1000 PRO microplate reader (Tecan, Switzerland). Following 24 h growth of the pre-treatment biofilm, and after 72 h of antibiotic treatment, Ebba680 fluorescence (Ex. λ 535 nm, Em. λ 660 nm) was recorded via a 15 × 15 area scan (circle-filled). Signals from columns 8–15 were compiled in GraphPad Prism 8 to quantify the biofilm as well as anti-biofilm effects of the antibiotics. Signals from columns 1–7 were excluded to avoid the Kirby-Bauer discs and the antibiotic-containing cavities in the agar.

### 2.11 Statistical analysis

Data was organized and processed with GraphPad Prism 8 (Graphpad Software, La Jolla, CA, USA).

### 2.12 Preparation and analysis of images

Fiji (Wisconsin, USA) [25,26] was used to process and perform orthogonal analysis of image stacks acquired by confocal microscopy, and to analyse the physical dimensions of biofilms in images collected by automated microscopy.

## 3 Results

### 3.1 Antibiotic susceptibility in different growth conditions

When selecting antibiotics to be studied for their possible effects on biofilm, we wanted a broad panel of representative agents from multiple antimicrobial classes, irrespective of the repertoire currently in clinical use. The antibiotics were (EUCAST abbreviations and classes in parenthesis): ampicillin (AMP, penicillins), oxacillin (OXA, penicillins), cefoxitin (CXI, cephamycins), vancomycin (VAN, glycopeptides), polymyxin B (polypeptides), rifampicin (RIF, rifamycins), ciprofloxacin (CIP, quinolones), erythromycin (ERY, macrolides), tetracycline (TET, tetracyclines) and gentamicin (GEN, aminoglycosides). To confirm that all antibiotic preparations showed antimicrobial activity as expected, we tested their efficacies in standard microbroth dilution assays. According to EUCAST guidelines, we used the ISO strain *E. coli* ATCC™ 8739™ grown in Mueller-Hinton II (M − H) broth. This generated the expected MIC values for all antibiotic preparations (Table 1).

**Table 1:** MIC determination of *E. coli* ISO strain ATCC™ 8739™ by microbroth dilution.

Target	Class	Antimicrobial agent	Number of tests	MIC (μg/mL)	
				Mode	Range
Cell wall	Penicillin	Ampicillin	6	4	4–8
		Oxacillin	7	256	128–256
	Cephalosporins	Cefoxitin	6	2	2
	Glycopeptide	Vancomycin	6	256	256
Cell membrane	Polypeptides	Polymyxin B	5	0.5	0.25–0.5
Nucleic acids	Rifamycin	Rifampicin	6	4	4–8
	Quinolones	Ciprofloxacin	8	<0.25	<0.25
Protein synthesis	Macrolides	Erythromycin	6	64	64
	Tetracyclins	Tetracycline	6	2	2–4
	Aminoglycosides	Gentamicin	6	1	1–2

**Fig. 1:**
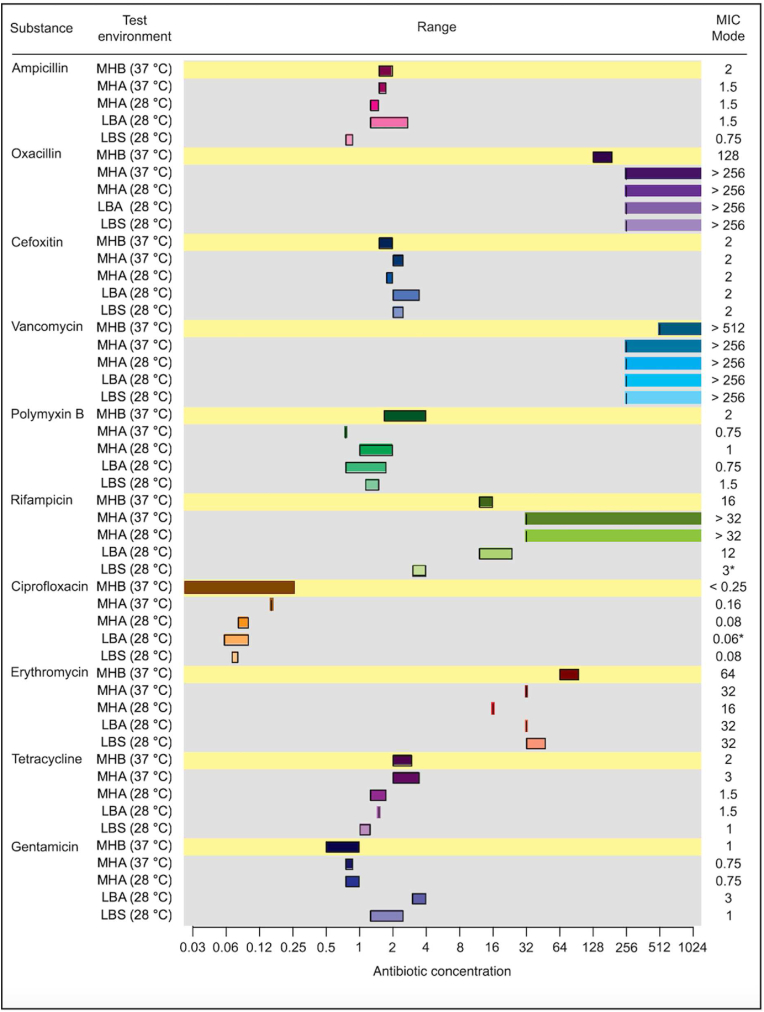
**Antibiotic susceptibility of *S.* Enteritidis strain 3934 at different growth conditions.** The microbroth dilution assay (yellow lines) was used to determine the minimum inhibitory concentration (MIC) of antibiotics in Mueller Hinton II (M − H) broth cultures (MHB) at 37 °C. The E-test (grey areas) was used to determine MIC of bacteria grown on the air-solid interface of M − H agar (MHA), LB agar (LBA), and LB without salt agar (LBS) at 37 °C and 28 °C. The range of MIC results for each antibiotic are indicated as coloured horizontal bars. Data is based on 3 experimental and 2 technical replicates. * = the lowest value in the event of more than 1 mode. (For interpretation of the references to colour in this figure legend, the reader is referred to the Web version of this article.)

As no standard strains are defined for susceptibility testing of bacterial biofilms, we used *Salmonella* Enteritidis (*S.* Enteritidis) strain 3934, a wild type strain known to present typical biofilm morphologies linked to its expression of the ECM components curli and cellulose when grown on Luria Bertani (LB) agar plates under hypoosmotic conditions [24]. Since this microbe and its biofilm-promoting assay conditions differ distinctly from current EUCAST-guided AST methodology, we started by analysing in a stepwise manner how altered growth conditions influenced the antibiotic efficacies on this strain. To define the baseline MIC for each antibiotic on *S.* Enteritidis 3934, we performed microbroth dilution assays with bacteria cultured in M − H broth at 37 °C (Fig. 1). Next, we tested if a shift from liquid culture (M − H broth) to solid conditions (M − H agar plates) influenced the antibiotic efficacies by peforming E-test on M − H agar at 37 °C. The agar-based MIC showed inhibition by AMP, CXI, polymyxin B, CIP, ERY, TET, and GEN. However, the results for OXA and RIF differed from those from liquid cultures, demonstrating that the switch from growth in liquid culture to growth on solid agar was associated with decreased MIC to the above tested antibiotic concentrations to 2 of the 10 antibiotics tested.

Since *Salmonella* primarily grow biofilm at 28 °C, we next analysed how the temperature influenced the AST by performing the E-test on M − H agar at 28 °C. A 2-fold decrease was observed for CIP, ERY, and TET, whereas other antibiotics only showed differences in the range of the reproducibility of the assay.

Since *Salmonella* routinely is cultured on Luria Bertani (LB) agar rather than M − H agar, we next tested the efficacies of antibiotics on *S.* Enteritidis 3934 growing on LB agar. The E-tests revealed a 2-fold increase for ERY and 4-fold increase for GEN. RIF gave an unexpected result, as the shift to LB agar restored *Salmonella's* susceptibility to this antibiotic. Other antibiotics only showed differences in the range of the reproducibility of the assay.

Finally, we performed the E-test under optimal *Salmonella* biofilm-inducing conditions using LB agar w/o salt at 28 °C. Compared to the LB agar, slight increases in MICs were observed for polymyxin B and CIP, minor decreases were detected for AMP, TET, and GEN, while CXI and ERY were unaffected. Interestingly, bacteria became clearly sensitized towards RIF under biofilm-inducing conditions, shown by a factor of 4. The sensitivity to RIF was even greater when compared to the liquid AST in M − H medium, showing a 5.3-fold difference.

### 3.2 Biofilm-AST by combined optotracing and disc diffusion methods

**Fig. 2:**
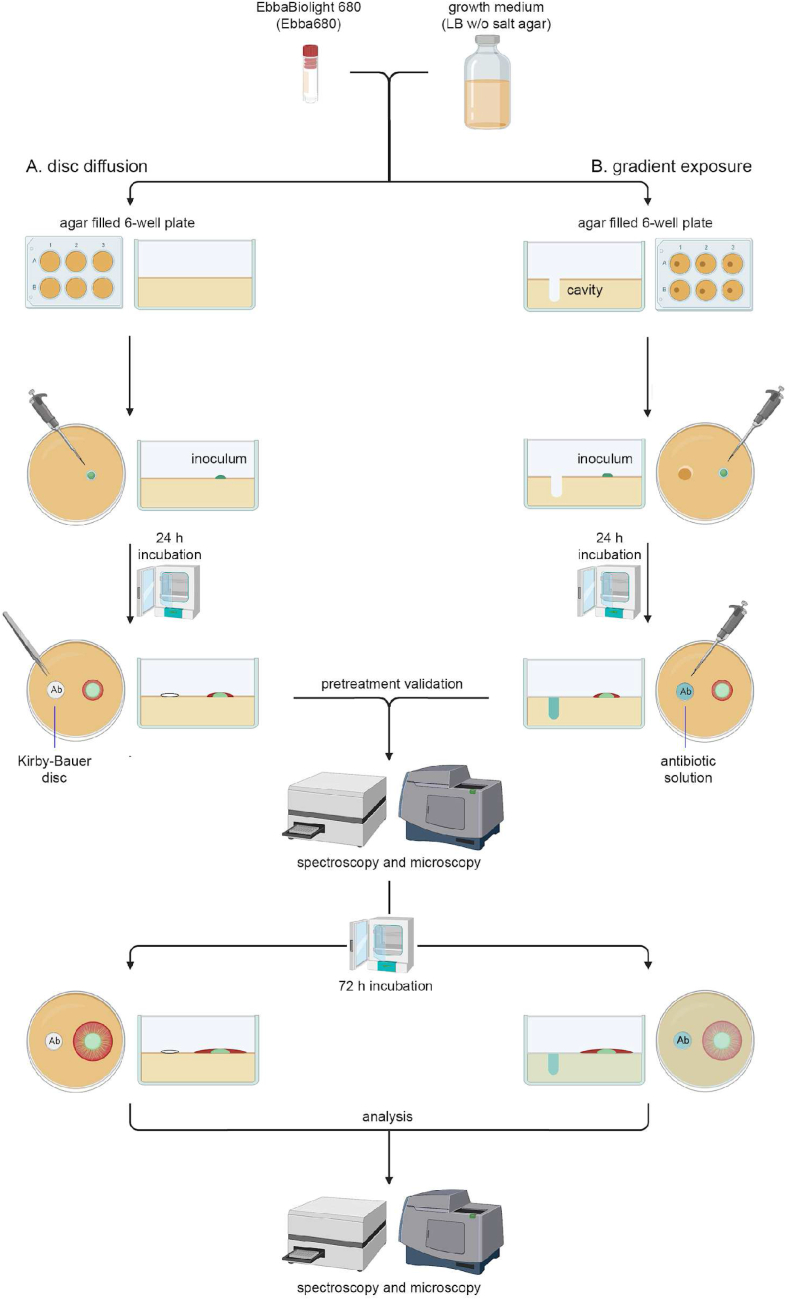
**Workflow of optotracer-based biofilm-ASTs.** The optotracer EbbaBiolight 680 (Ebba680) is added to biofilm-promoting nutrient agar, here LB w/o salt agar, which is added to the wells of 6-well plates. (**a**) To perform disc diffusion tests, 10 μl inoculum from a GFP-expressing *Salmonella* culture in exponential phase is placed on top of the agar 10 mm off-centre in each well. Following incubation at 28 °C for 24 h, formation of pre-treatment biofilms is validated by automated fluorescence microscopy and pre-treatment data is collected. Phase contrast images are used to measure the physical dimensions of the biofilm, and the amount of curli ECM is quantified by spectrophotometric recordings. Antibiotic-containing Kirby-Bauer discs are then placed 10 mm from the centre of pre-treatment biofilms using blank discs as control. Following incubation at 28 °C for 72 h, end-stage analysis of the biofilms are performed as described in for the pre-treatment validation. (**b**) To perform gradient exposure for dose-response analysis, a cavity is created in the Ebba680-supplemented agar, positioned 10 mm off-centre in the 6-well plates. The experimental procedure for pre-treatment and end-stage analysis follows same process as in (**a**), except that antibiotic exposure is initiated by addition of 80 μl antibiotic in solution to the cavities. Each 6-well plate contains 5 different concentrations of one antibiotic, and one blank control.

**Fig. 3:**
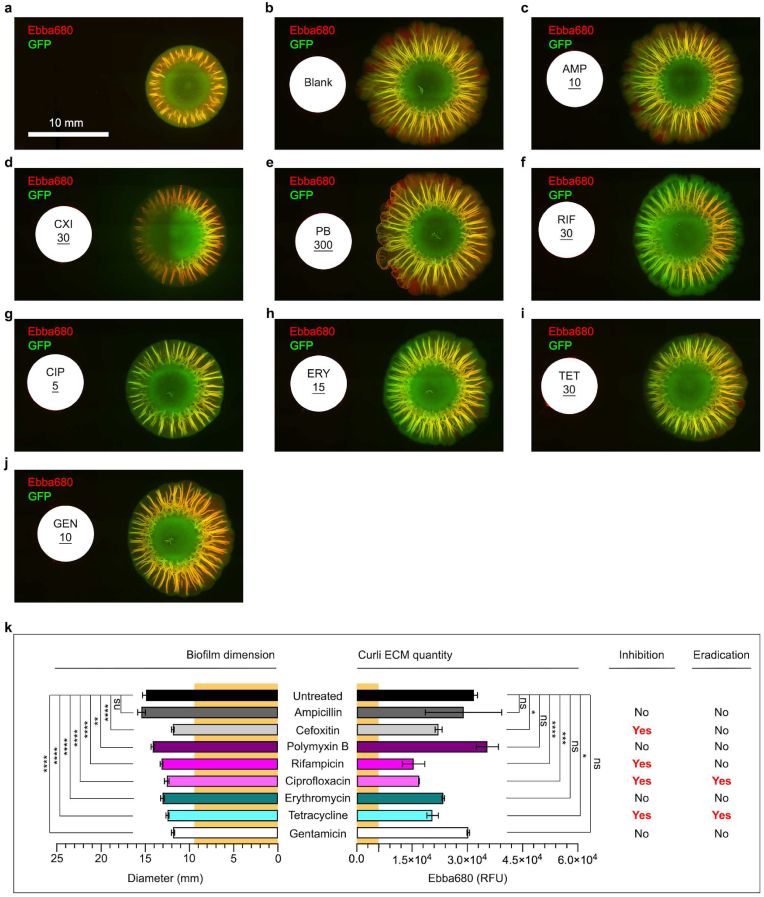
**Antibiotic susceptibility of *S.* Enteritidis biofilms**. Fluorescence micrographs of *S.* Enteritidis 3934-p2777 showing the biofilm formed on LB agar w/o salt supplemented with EbbaBiolight 680 at (**a**) 24 h, and (**b-j**) after an additional 72 h growth next to discs containing (**b**) no antibiotic (Blank), (**c**) ampicillin (AMP), (**d**) cefoxitin (CXI), (**e**) Polymyxin B, (**f**) rifampicin (RIF), (**g**) ciprofloxacin (CIP), (**h**) erythromycin (ERY), (**i**) tetracycline (TET), and (**j**) gentamicin (GEN). The low-copy number plasmid p2777 enables stable GFP expression and confers resistance to ampicillin. Representative images from 3 experimental repeats are shown. (**k**) Effects of antibiotics on the diameter (mm) and the quantity of curli ECM, based on fluorescence (RFU) from Ebba680 bound to curli, on *S.* Enteritidis 3934 biofilms. The diameter (9.5 mm) and curli ECM quantity (5926 RFU) of the pre-treatment biofilm in (**a**) is indicated across all antibiotics by yellow background. Antibiotics' effect on biofilm inhibition and eradication is indicated by Yes/No. Data is based on 3 experimental replicates and 2 technical repeats. P-values < 0.05, <0.01, <0.001 and < 0.0001 are indicated as *, **, *** and ****, ns = non-significant. (For interpretation of the references to colour in this figure legend, the reader is referred to the Web version of this article.)

To study an antibiotic's efficacy on biofilm bacteria, one must ensure that bacteria, originating from a planktonic inoculum, have entered the biofilm lifestyle prior to antibiotic exposure. To develop an AST specifically designed for the biofilm lifestyle of *Salmonella*, we devised a workflow based on our recently reported optotracer-based semi-high throughput biofilm assay. In this assay, growth of *Salmonella* biofilms can be monitored in real-time by fluorescence imaging and spectroscopy as the biofilm forms on agar supplemented with the optotracer EbbaBiolight 680 (Ebba680) [17]. To translate this live biofilm detection assay into a biofilm-AST, we combined the optotracer-based assay and the Kirby-Bauer disc diffusion method (Fig. 2a). Since optotracing is a fluorescence-based assay, we used *Salmonella* 3934-p2777, an isogenic strain harbouring a GFP-expressing plasmid that makes cells visible under fluorescence. According to the published protocol, we inoculated 10 μL from a culture of *S.* Enterica 3934-p2777 onto Ebba680-supplemented LB agar w/o salt cast in the wells of 6-well plates. Following incubation at 28 °C for 24 h, we analysed if biofilm had formed using an automated microscope. Fluorescence imaging of the macro-colony revealed an organized biofilm with a central core and an intermediate region as previously described [17], and the distinct fluorescence from GFP-expressing bacteria varied in intensity relative to the entire biofilm (Fig. 3a). In the red channel, fluorescence of Ebba680 bound to curli showed this ECM component to form distally projecting radial ridge patterns and channel-like structures typical to biofilms by this strain [17]. Since this verified that *Salmonella* had formed a biofilm, we applied this analysis in all following experiments to ensure that bacteria had adopted a biofilm lifestyle before we initiated antibiotic exposure. Prior to antibiotic exposure, we also collected data of the status of the pre-treatment biofilms (Fig. 3a, k). Analysis of the fluorescence images showed that biofilms had grown to a diameter of 9.5 mm. The amount of ECM, assessed by spectrophotometric recordings (Ex. λ 530 nm, Em. λ 660 nm) of curli-bound Ebba680 fluorescence, amounted to 5926 relative fluorescence units (RFU).

Once the pre-treatment characteristics of the biofilm were determined, we initiated antimicrobial treatment using commercially available Kirby-Bauer discs containing AMP, CXI, polymyxin B, RIF, CIP, ERY, TET, and GEN (Fig. 2a). Discs with no antibiotics were used to mock antimicrobial treatment. In each well, one disc was placed on the surface of the agar 1 cm away from the periphery of the pre-treatment biofilm. Plates were then incubated at 28 °C for 72 h, to give time for the antibiotics to diffuse from the disc through the agar and to exert their effects on the growing biofilm. At the incubation endpoint, we placed the 6-well plate in the automated microscope to visualize the morphology and determine the size of the treated biofilm. Also, we quantified the amount of curli ECM based on fluorescence from the curli-bound Ebba680. GFP and Ebba680 fluorescence revealed unabated growth in the mock treated biofilm (Fig. 3b). This was shown as an enlargement of the biofilm, expansion of the intermediate region, and elongation of the curli-rich ridge patterns and channel-like structures of the ECM. The biofilm had a diameter of 14.9 mm, representing an increase of 157% compared to the pre-treatment biofilm (Fig. 3k).

The curli-bound Ebba680 fluorescence was 31822 RFU, corresponding to 537% increase of curli ECM as the biofilm grew from the pre-treatment (5926 RFU) stage. AMP treated biofilms showed similar growth patterns and biofilm morphologies as the mock treated biofilm (Fig. 3c). A diameter of 15.4 mm and curli-bound Ebba680 fluorescence of 29013 RFU showed that the size and curli ECM quantity were similar in the AMP and mock treated biofilm (Fig. 3k). The lack of inhibitory effects of AMP was un-surprising, since this strain harbours a plasmid encoding AMP resistance for selection of GFP expressing bacteria.

Biofilms developed when treated by CXI showed markedly different morphology compared to the mock treated. Fluorescence imaging of this biofilm showed reduced GFP signal in the area facing the antibiotic disc, possibly indicating reduced bacterial viability at this site (Fig. 3d). CXI treatment resulted in a biofilm of smaller size, showing a diameter of 11.9 mm (Fig. 3k). Quantification of curli ECM by Ebba680 yielded 22216 RFU, which confirmed the visually observed reduction of the curli-rich intermediate region compared to mock treated biofilm. This suggests that CXI inhibits biofilm growth as well as ECM development. However, the diameter showed 157% increase compared to the pre-treatment biofilm, suggesting that a lag time exists between initiation of CXI treatment and the onset of visible effects. This lag time likely represents the time for diffusion of CXI through the agar. To assess if the biofilm contained any viable cells post-treatment, we adopted a binary analysis by harvesting the entire biofilm which we used to inoculate fresh antibiotic free M − H broth. The turbidity observed after 16 h incubation at 37 °C clearly demonstrated a viable bacterial culture. Collectively, this shows that CXI was unable to eradicate the biofilm and do not have biofilm-cidal effects.

Polymyxin B treated biofilm did not show any visible size reduction compared to the mock treated, however, statistical analysis revealed that the diameter (14.2 mm) was slightly smaller post-treatment (Fig. 3e, k). The ECM, shown as a curli-rich intermediate region with distally projecting ridges and channel-like structures, appeared in the same quantity as in mock treated biofilms. Careful inspection of the red fluorescence from Ebba680 revealed, however, that new continuous wall structures had formed along the periphery of the biofilm facing the Polymyxin B disc. The appearance of these curli rich, barrier-like structures suggest that Polymyxin B does not inhibit biofilm development *per se*, rather it may induce a resistance response by means of increased curli ECM production. The bacterial viability test post-treatment showed growth, which suggested a lack of biofilm-cidal effects of this antibiotic.

RIF treatment resulted in a smaller biofilm size (13.2 mm diameter) compared to the mock treated, a reduction most prominent in the intermediate region facing the RIF disc (Fig. 3f, k). While distally projecting ridges and channel-like structures were present, Ebba680 fluorescence showed reduced amount of curli throughout the entire biofilm. This was verified by spectrophotometric recordings, in which the curli-bound Ebba680 signal (15441 RFU) was only 48% of that in the mock treated biofilm. The significant reduction of the biofilm size and curli ECM quantity suggests that RIF has biofilm inhibitory effects. Assessment of biofilm viable cells post-treatment showed bacterial growth, which implies that RIF lacks biofilm-cidal effects.

CIP treatment reduced both the biofilm size and the curli rich intermediate region (Fig. 3g). With a diameter of 12.6 mm and Ebba680 signal of 16974 RFU, both measurements were significantly lower (15% and 53% respectively) than those of the mock treated (Fig. 3k). This lead us to conclude that CIP has biofilm inhibitory effects. The CIP treated biofilm had however, grown compared to the pre-treatment biofilm, indicating a lag time between initiation of CIP treatment and the onset of inhibitory effects. When assessing biofilm viable cells post-treatment, no growth of the culture was observed. This implied that CIP in addition to inhibitory effects also has biofilm-cidal activity.

ERY treatment generated a reduced biofilm size, a diameter of 13.1 mm was significantly smaller than the mock treated biofilm (Fig. 3h, k). Ebba680 fluorescence showed normal development of the curli-rich intermediate region, albeit the intensity of the red fluorescence (23502 RFU) was weaker throughout the biofilm compared to the mock treated. Statistical analysis showed however lack of significance, implying that ERY has no biofilm inhibitory effects. Assessment of biofilm viable cells post-treatment showed growth, suggesting that ERY also lacks biofilm-cidal activity.

TET treatment led to a reduced biofilm size (diameter 12.5 mm) compared to the mock treated control (Fig. 3i, k). Fluorescence from Ebba680-bound curli showed the characteristic curli-rich distally projecting ridges and channel-like structures, however, a thinner intermediate region was observed that was reflected in a lower signal intensity (20588 RFU) compared to the mock treated. This suggests that TET has biofilm inhibitory effects. The TET treated biofilm had however, grown in size compared to the pre-treatment biofilm, suggesting that a lag time exists between the application of the TET disc and the onset of inhibitory effects. Analysis of biofilm viable cells post-treatment showed no growth. Collectively, this implied that TET exert inhibitory as well as biofilm-cidal effects.

GEN treatment caused a reduced size (11.9 mm diameter) compared to the mock treated biofilm (Fig. 3j and k). The reduction was most prominent in the intermediate region facing the GEN disc. Despite this, strong Ebba680 fluorescence (30261 RFU) indicated a rich presence of curli throughout the distally projecting ridges and channel-like structures of the ECM. As the Ebba680 signal was equal to the signal in mock treated biofilms, it suggests that GEN lacks biofilm inhibitory effects. Assessment of biofilm viable cells post-treatment showed growth, suggesting that GEN also lack biofilm-cidal activities.

### 3.3 Gradient exposure for dose-response determination in the biofilm-AST

To overcome the restriction caused by the fixed amount of antibiotics in the Kirby-Bauer discs, we modified the mode of antibiotic administration in our optotracing-based biofilm-AST. As we casted Ebba680-supplemented LB w/o salt agar in 6-well plates, we created a cavity in the agar of each well to which antibiotics in solutions later were added (Fig. 2b). We inoculated GFP-expressing *Salmonella* strain 3924-p2777 on top of the agar, 10 mm away from the cavity, and incubated the plate at 28 °C to allow biofilms to form. After 24 h, we collected pre-treatment data by visualizing the morphology, determining the size, and quantifying the amount of curli ECM in the biofilms. We then initiated the dose-response assay by adding antibiotics in solution to the cavities in the agar of each well. One antibiotic was used per 6-well plate at 5 different concentrations, leaving one well for comparison to biofilm growth without antibiotic exposure. Based on results from the optotracing-based biofilm-AST using Kirby-Bauer discs, we selected 4 antibiotics to be tested: CIP which was the most effective anti-biofilm antibiotic; RIF which caused a reduction in the ECM production; CXI which caused a change in GFP; and Polymyxin B to which bacteria became resistant most likely due to increased ECM production. Following addition of antibiotics to the cavities of each well, the 6-well plates were incubated for 72 h, then we ended the experiment and analysed the effect of the antibiotics on the biofilms by automated microscopy and by spectroscopy.

**Fig. 4:**
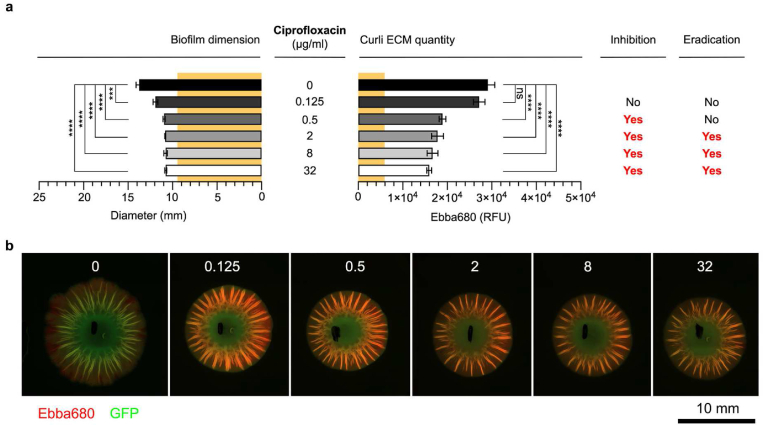
**Ciprofloxacin dose-response biofilm-AST of *S.* Enteritidis biofilm.** (**a**) Effects of ciprofloxacin on the diameter (mm) and the curli ECM quantity measured by fluorescence (RFU) from Ebba680 bound to curli in *S.* Enteritidis 3934-p2777 biofilm. The low-copy number plasmid p2777 enables stable GFP expression and confers resistance to ampicillin. The diameter (9.5 mm) and curli ECM quantity (5926 RFU) of the pre-treatment biofilm is indicated across all antibiotics by yellow background. Biofilm inhibitory or eradication effects are indicated by Yes/No. Data is based on 3 experimental replicates with 2 technical repeats. P-values < 0.001 and < 0.0001 are indicated as *** and ****, ns = non-significant. (**b**) Fluorescence micrographs show the effects of each antibiotic dose on the biofilm. Images were collected by automated microscopy. (For interpretation of the references to colour in this figure legend, the reader is referred to the Web version of this article.)

CIP was applied at 0.125–32 μg/mL in the cavity next to the pre-formed biofilms. Compared to the endpoint size of mock treated biofilm, CIP significantly reduced biofilm dimension already at the lowest concentration (Fig. 4a and b). The diameter further decreased at 0.5 μg/mL to a size that remained also when exposed to higher concentrations. Based on Ebba680 fluorescence, the amount of curli ECM was unaffected at 0.125 μg/mL CIP despite reductions in biofilm diameter. However, significant reduction of curli ECM was observed at 0.5 μg/mL, to an amount that remained also at the higher concentrations. To assess the dose at which biofilm formation was inhibited, we introduced the concept “minimal dose-response inhibitory concentration” (MD-RIC). The new expression was needed since biofilm cells are exposed to a time-dependent gradient of antibiotics diffusing from the cavity through the agar, rather than a defined concentration to which cells are immediately exposed when performing “minimal biofilm inhibitory concentration” (MBIC) and “minimum biofilm eradication concentration” (MBEC) determinations. Using curli detected by Ebba680 as basis for assessment, we identified the MD-RIC at between 0.125 and 0.5 μg/mL. When evaluating biofilm viable cells, growth was observed at 0, 0.125 and 0.5 μg/mL, which ceased in biofilms treated with 2 μg/mL CIP and above. In accordance to the above-mentioned nomenclature, our data implied that the minimal dose-response eradication concentration (MD-REC) of CIP was between 0.5 and 2 μg/mL.

**Fig. 5:**
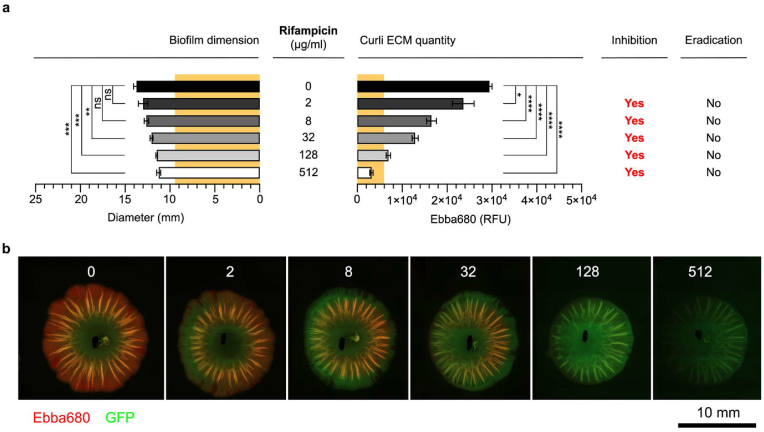
**Rifampicin dose-response biofilm-AST of *S.* Enteritidis biofilm.** (**a**) Effects of rifampicin on the diameter (mm) and the curli ECM quantity measured by fluorescence (RFU) from Ebba680 bound to curli in *S.* Enteritidis 3934-p2777 biofilm. The low-copy number plasmid p2777 enables stable GFP expression and confers resistance to ampicillin. The diameter (9.5 mm) and curli ECM quantity (5926 RFU) of the pre-treatment biofilm is indicated across all antibiotics by yellow background. Biofilm inhibitory or eradication effects are indicated by Yes/No. Data is based on 3 experimental replicates with 2 technical repeats. P-values < 0.05, <0.01, <0.001 and < 0.0001 are indicated as *, **, *** and ****, ns = non-significant. (**b**) Fluorescence micrographs show the effects of each antibiotic dose on the biofilm. Images were collected by automated microscopy. (For interpretation of the references to colour in this figure legend, the reader is referred to the Web version of this article.)

RIF, found in the disc diffusion to be biofilm inhibitory but not biofilm-cidal, was used at 2–512 μg/mL in the modified biofilm-AST. A significant reduction in the biofilm diameter was observed at 32 μg/mL, and this trend continued with each increase in antibiotic concentration (Fig. 5a and b). However, the curli ECM amount was significantly reduced already when exposed to 2 μg/mL RIF. Each increase in RIF corresponded with a decrease in curli ECM to the extent that the presence of 128 μg/mL RIF reduced the curli ECM of the treated biofilm to the same quantity as observed in the pre-treatment biofilm at day 1. Subsequent RIF increase to 512 μg/mL reduced the curli ECM to an even lower level, implying strong inhibitory effects of this antibiotic on ECM production. We concluded that the MD-RIC of RIF was ≤2 μg/mL. When evaluating biofilm viability by re-inoculating the biofilm colony into new antibiotic free M − H broth, growth occurred in all cultures. Thus, no MD-REC for RIF could be identified.

**Fig. 6:**
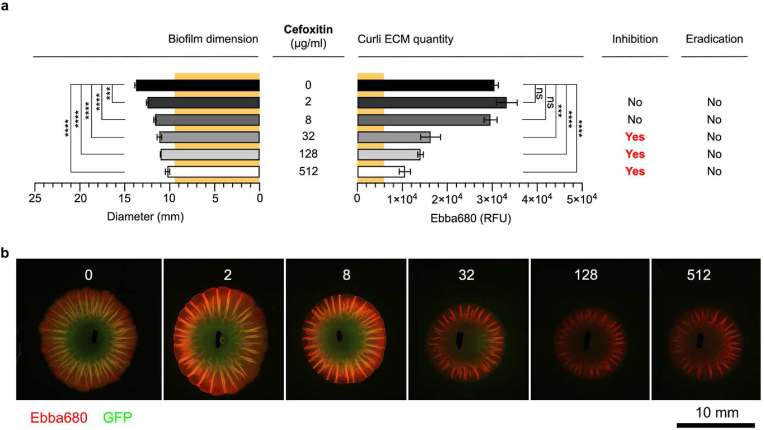
**Cefoxitin dose-response biofilm-AST of *S.* Enteritidis biofilm.** (**a**) Effects of cefoxitin on the diameter (mm) and the curli ECM quantity measured by fluorescence (RFU) from Ebba680 bound to curli in *S.* Enteritidis 3934-p2777 biofilm. The low-copy number plasmid p2777 enables stable GFP expression and confers resistance to ampicillin. The diameter (9.5 mm) and curli ECM quantity (5926 RFU) of the pre-treatment biofilm is indicated across all antibiotics by yellow background. Biofilm inhibitory or eradication effects are indicated by Yes/No. Data is based on 3 experimental replicates with 2 technical repeats. P-values < 0.001 and < 0.0001 are indicated as *** and ****, ns = non-significant. (**b**) Fluorescence micrographs show the effects of each antibiotic dose on the biofilm. Images were collected by automated microscopy. (For interpretation of the references to colour in this figure legend, the reader is referred to the Web version of this article.)

CXI, which the disc diffusion assay showed to inhibit biofilm formation but not eradicate biofilm bacteria, was used at 2–512 μg/mL in the modified biofilm-AST. At 2 μg/mL, a significant reduction in biofilm dimension occurred, and each increase in antibiotic concentration resulted in a corresponding decrease of the size (Fig. 6a and b). Yet, the curli-bound Ebba680 fluorescence showed no reduction in the biofilm's curli ECM in the presence of 2 and 8 μg/mL CXI despite the effectiveness of these concentrations in reducing the biofilm size. Significant reduction of the curli ECM was only observed at 32 μg/mL and above. We thus concluded that the MD-RIC of CXI was 8 ≤ 32 μg/mL. When evaluating biofilm viable cells, we observed growth in all cultures irrespective of CXI treatment. Thus, no MD-REC for CXI could be identified.

**Fig. 7:**
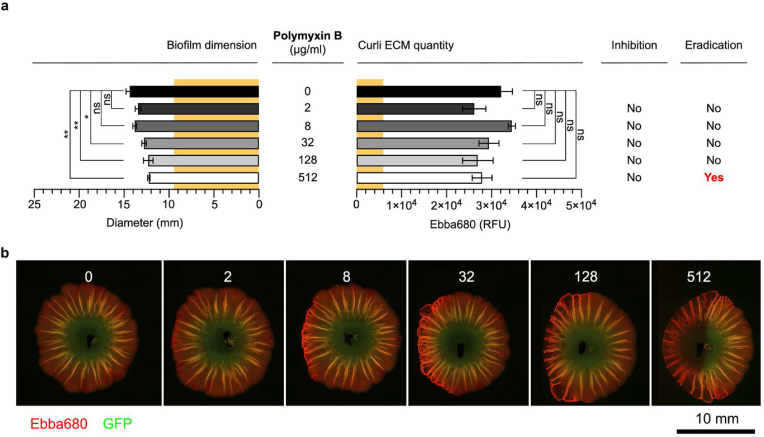
**Polymyxin B dose-response biofilm-AST of *S.* Enteritidis biofilm.** (**a**) Effects of polymyxin B on the diameter (mm) and the curli ECM quantity measured by fluorescence (RFU) from Ebba680 bound to curli in *S.* Enteritidis 3934-p2777 biofilm. The low-copy number plasmid p2777 enables stable GFP expression and confers resistance to ampicillin. The diameter (9.5 mm) and curli ECM quantity (5926 RFU) of the pre-treatment biofilm is indicated across all antibiotics by yellow background. Biofilm inhibitory or eradication effects are indicated by Yes/No. Data is based on 3 experimental replicates with 2 technical repeats. P-values < 0.05 and < 0.01 are indicated as *, and **, ns = non-significant. (**b**) Fluorescence micrographs show the effects of each antibiotic dose on the biofilm. Images were collected by automated microscopy. (For interpretation of the references to colour in this figure legend, the reader is referred to the Web version of this article.)

Polymyxin B was identified by disc diffusion as the only antibiotic having a contributory effect on curli ECM production. As we treated the biofilms with 2–512 μg/mL of polymyxin B in the modified biofilm-AST, a significant reduction in biofilm diameter occurred at 32 μg/mL, and minor further reductions were observed at 128 and 512 μg/mL (Fig. 7a and b). Curli-bound Ebba680 fluorescence showed no significant changes in the biofilm's curli ECM across the tested concentrations. No MD-RIC of polymyxin B could thus be identified within the range used in this assay. In the biofilm viability test, growth was observed in all cultures grown from biofilms treated with 2–128 μg/mL polymyxin B. Interestingly, no growth was observed in the culture grown from biofilms treated with 512 μg/mL polymyxin B. This implied a MD-REC of 128–512 μg/mL polymyxin B.

Taken together, the dose-response mode of antibiotic administration in our modified optotracer-based biofilm-AST identified the MD-RIC and MD-REC of CIP, revealing both biofilm-inhibitory and biofilm-cidal effects of this antibiotic on *Salmonella* biofilms. For RIF and CXI, our assay revealed no eradication of biofilm bacteria despite obvious inhibitory effects. This implied a disconnect between inhibition and eradication activities. This was further exemplified in the case of polymyxin B, where we surprisingly showed that polymyxin B has biofilm eradication effects despite the lack of biofilm inhibitory effects.

### 3.4 Susceptibility of *Salmonella* to antibiotics is lifestyle dependent

With a method at hand by which we could determine the MD-RIC and MD-REC of antibiotics acting on biofilm bacteria, we investigated if the curli component of the ECM influences the antibiotic susceptibility of the biofilm. We repeated the gradient exposure for dose-response determination of CIP, RIF, CXI, and polymyxin B in the biofilm AST, this time using *S.* Enteritidis 3934 *ΔcsgA*-p2777. This strain, which is isogenic to *S.* Enteritidis 3934-p2777, lacks curli production. The MD-REC was for CIP ≤0.125 μg/mL, CXI 128–512 μg/mL, and polymyxin B 128–512 μg/mL, while no bactericidal concentration was observed for RIF. The appearance of susceptibility to CXI and the decreased MD-REC of CIP in biofilms lacking curli suggest a role of curli ECM in conferring resistance to these two antibiotics.

We next investigated how the bacterial lifestyle influences the antimicrobial activities. Proper comparison of the antibiotic susceptibility of planktonic and biofilm cells requires that an equal number of cells are used in the planktonic test culture as in the pre-treatment biofilms formed during growth for 24 h prior to antibiotic exposure. Following homogenization, viable counts of the pre-treatment biofilms showed a density of circa 10^9^ CFU/mL. We thus prepared fresh liquid cultures of *S.* Enteritidis strain 3934-p2777 at this cell density, which we used when examining the minimum bactericidal concentration (MBC) of CIP, RIF, CXI and polymyxin B on planktonic cells. By applying a range of concentrations of each antibiotic in microbroth dilution assays, the MBC was determined by analysing cell viability of treated bacteria re-inoculated in antibiotic free medium. The resulting MBC was for CIP 0.5 μg/mL, CXI 8 μg/mL, and polymyxin B 32 μg/mL, while RIF showed no bactericidal concentration (Table 2). By comparing the MBC of planktonic cells to the MD-REC of the optotracer-based biofilm-AST, up to 4-fold reduction of MBC was observed for CIP, and 16-fold for polymyxin B. Interestingly, CXI showed bactericidal activity in the planktonic culture, while it had no effect on the biofilm. Collectively, this suggests that bacteria in biofilm are less sensitive to some antibiotics (CIP, polymyxin B) compared to the planktonic state. For other antibiotics (CXI) the biofilm offers complete protection, making the antibiotic totally ineffective.

**Table 2:** Antibiotic susceptibility of sessile (biofilm) and planktonic bacteria.

	Biofilm MD-REC^a^ (μg/mL)	Planktonic MBC^b^ (μg/mL)	Ratio MD-REC/MBC
CIP	0.5–2	0.5	1–4
RIF	-c	–	–
CXI	–	8	–
Polymyxin B	128–512	32	4–16

a MD-REC = minimal dose-response eradication concentration.

b MBC = minimum bactericidal concentration.

c No MD-REC or MBC detected within the concentrations tested.

## 4 Discussion

The biofilm antibiotic susceptibility test reported here, the optotracer-based biofilm-AST, specifically analyses the effects of antimicrobials on biofilm bacteria. Key to this assay is the optotracer Ebba680 present in the agar on which biofilms form. Unlike conventional fluorophores, the fluorescent signal from this non-toxic tracer molecule remains off until the biomolecular binding target appears [17]. This binding-induced on-switch of fluorescence enables live, dynamic studies in which the optotracer continuously reports the growth and/or eradication of the biofilm in real-time. In *Salmonella* biofilms, Ebba680 acts as an *in situ* reporter of curli in the ECM of biofilms forming on LB w/o salt agar [17]. Detection of the essential ECM component curli defines the method as biofilm specific. Yet a critical feature is the non-destructive nature of optotracing. This allows for pre-treatment assessment of the biofilm prior to antimicrobial exposure. Methods for pre-treatment assessment that complement end stage analysis are considered essential for future improvement of the clinical validity of anti-biofilm assays [27].

The optotracing-based biofilm-AST facilitates analysis of new parameters, which we believe are an important complement to the commonly used minimum biofilm inhibitory/eradication concentration (MBIC/MBEC). While MBIC and MBEC solely centre around the bacterial cells, the optotracing-based biofilm-AST enables visualization of the biofilm morphology by live microscopy, determination of the biofilm's physical dimensions, and quantification of the ECM reflected by the amount of curli. We compiled a general overview on the effect of antimicrobials on these novel aspects of *Salmonella* biofilms by including a wide range of compounds from different antibiotic classes in our screen. Our data showed that antibiotic pressure is almost always linked to a significant reduction in the biofilm dimension. It was interesting to notice, however, that the smaller dimension is not always mirrored by a reduction in the curli ECM quantity, as was found for polymyxin B and GEN. This observation hints at the known mechanism of biofilm attributed AMR whereby the amount of curli increases in response to antibiotic pressure [[28], [29], [30]]. As we formulated a definition based on the curli ECM quantities in treated versus non-treated biofilms of the same age, we identified several antibiotics effective in inhibiting curli ECM formation in *Salmonella* biofilms, such as CXI, RIF, CIP, and TET. Among these, CIP and RIF were highly effective antibiotics, and the latter was able to reverse the trend of curli ECM formation to levels lower than that of the pre-treatment biofilms. This opens for a novel use of RIF, and possibly other rifamycins, as therapeutics to counteract ECM formation. In combination therapies, RIF may thus increase the susceptibility of biofilm bacteria to other antibiotics. Another interesting observation was that while TET and GEN inhibited biofilm dimension to roughly the same degree, TET exerted a substantially stronger inhibition of the curli ECM production. This demonstrates the importance of using multiple parameters when studying antibiotic activities in biofilms.

In *Salmonella* and *E. coli*, the expression of curli alongside other ECM materials such as cellulose, determines the biofilm's morphology and the majority of encompassing micro- and macrostructures, giving it tissue-like properties [[31], [32], [33]]. It is reported that curli assemble into amyloid fibers which subsequently form basket-like structures around cells in the upper layer of colonies [34,35]. As a major architectural element, curli has a direct effect on the porosity, density, water content, and absorption properties of the biofilm [36]. Our data, demonstrating that the overall antibiotic susceptibilities of the biofilm, here shown by CIP and CXI, is influenced in a curli-deficient strain may thus not be surprising. Moreover, disruption of the curli ECM by antibiotics such as RIF may increase the susceptibility of biofilms to other antibiotics and become useful in combination therapies to effectively eradicate infections while at the same time reducing the overall use of antibiotics.

As the optotracer-based biofilm-AST allows the evaluation of multiple anti-biofilm effects directly on growing biofilms, it is poised to become a useful addition to the toolbox for biofilm-related studies. By complementing the MBEC Assay® (formerly known as the Calgary Biofilm Device), which determines the antimicrobials’ efficacy against biofilms growing at the liquid-solid interface, the optotracer-based biofilm-AST provides a means to evaluate air-solid interface biofilms. Together, these assays allow comprehensive assessment of a greater range of biofilm types than currently possible. This is further supported by the flexibility of the biofilm-AST, in which the growth conditions can be tailored to optimise biofilm-formation of the strain under study. Here, we used biofilm-forming strain *S.* Enteritidis 3934 as reference due to its well validated expression of amyloid curli fibres, rather than the conventional ISO strain *E. coli* ATCC™ 8739™. Assessing the applicability of the optotracer-based biofilm-AST for other bacterial species, possibly using a wider range of optotracers, will be intriguing subjects of future studies that hopefully contributes to an improvement of the clinical validity of future anti-biofilm assays.

## 5 Conclusion

This work pioneers a method that enables analysis of multiple effects antibiotics may have on bacterial biofilm formation. In addition to bacterial viability, the biofilm-AST visualizes effects on the biofilm morphology and allows quantification of biofilm dimensions prior to, during, and at the end of treatment. Importantly, selective tracing of the ECM component curli provides a quantitative measure on the antibiotics’ effect on this biofilm-specific macromolecular assembly. By putting the spotlight on the ECM, we envision that the biofilm-AST will give new insights of importance for future development of biofilm-specific antibiotics and adjuvant antibiotics alike, eventually improving the treatment outcome of biofilm infections.

## Data availability

The authors declare that data supporting the findings of this study are available upon reasonable request.

## Additional information

No Supplementary Information.

## CRediT authorship contribution statement

**Johannes A. Eckert:** Investigation, Methodology, Formal analysis. **Ming Rosenberg:** Investigation, Methodology, Formal analysis. **Mikael Rhen:** Writing – review & editing. **Ferdinand X. Choong:** Conceptualization, Supervision, Formal analysis, Data curation, Visualization, Project administration, Writing – original draft, Writing – review & editing. **Agneta Richter-Dahlfors:** Conceptualization, Formal analysis, Writing – original draft, Writing – review & editing, Project administration, Funding acquisition, Resources.

## Declaration of competing interest

The authors declare the following financial interests/personal relationships which may be considered as potential competing interests: Agneta Richter-Dahlfors reports a relationship with Ebba Biotech AB that includes: board membership and equity or stocks. Ferdinand X. Choong reports a relationship with Ebba Biotech AB that includes: equity or stocks. Agneta Richter-Dahlfors has patent issued to Richter Life Science Development AB. Agneta Richter-Dahlfors has patent pending to Richter Life Science Development AB. Ferdinand X. Choong has patent issued to Richter Life Science Development AB. Ferdinand X. Choong has patent pending to Richter Life Science Development AB.
